# ‘Probably just sexism’- gendered experiences of resource access in rugby

**DOI:** 10.1371/journal.pone.0303972

**Published:** 2024-05-21

**Authors:** Freja J. Petrie, Kelly A. Mackintosh, Chelsea Starbuck, Melitta A. McNarry

**Affiliations:** Department of Applied Sports, Technology, Exercise and Medicine, Swansea University, Swansea, Wales; IIPS: International Institute for Population Sciences, INDIA

## Abstract

Research pertaining to the experiences of women in rugby is scarce, which, coupled with the limited visibility of the sport and difficulty accessing resources, suggest that women’s rugby remains undervalued. Indeed, evidence of such gender inequalities remains largely anecdotal, with little rigorous research undertaken to understand the perspectives of women in rugby. This study aimed to explore the experiences of a diverse cohort of rugby players in relation to their participation in the sport and their ability to access resources. Twenty UK-based rugby players (10 men, 9 women and 1 non-binary person aged 29.1 ± 8.3 years) from school, university, club, military, and semi-professional environments, volunteered to participate in semi-structured interviews (36 ± 12 minutes) discussing their rugby experiences in relation to their gender and playing level. Interviews were transcribed verbatim, and a reflexive thematic analysis was undertaken. A widespread under-prioritisation of women in rugby was highlighted. Gender biases were apparent in access to changing rooms, pitches, quality coaches, and playing opportunities, and were reportedly propagated at the managerial level. Irrespective of gender, some amateur players reported difficulty accessing a suitable rugby environment. Insufficient player numbers precluded the formation of second teams, often resulting in inexperienced players competing beyond their ability. Women’s rugby players experienced considerable gender bias. This exploratory study highlights a need to address such issues to protect player welfare. Interventions to change the culture in rugby clubs and increased representation of women in managerial positions in rugby are recommended to enact meaningful change.

## Introduction

Rugby union is a field-based contact sport played by 2.7 million women worldwide, accounting for over a quarter of the total playing population [1]. Although such growth of the women’s game is positive, rugby’s longstanding male-dominated history may mean players currently experience gendered treatment during their engagement with the sport. The term gendered is used to describe a circumstance or characteristic that relates to, or is experienced by, people of one particular gender [2]. Gender typically refers to how a person chooses to identify, often in relation to ever-changing and culturally-specific societal norms of appearance and behaviour [3]. Examples of gender identities include man, woman, non-binary and genderfluid [3].

Whilst most evidence remains anecdotal, there are widespread reports of gendered treatment in sport [4–10], whereby women’s teams are generally less able to access resources whilst being undervalued by club and school management [4, 6, 8, 11, 12]. This was exemplified by Leahy et al. [11] who reported that boy’s teams in rugby-playing Irish schools were given precedence for both strength and conditioning, and injury-prevention efforts. Furthermore, girls had fewer opportunities to play rugby, with only 13% of the schools having a girls’ team despite 62% of the schools being co-ed [11]. Similarly, in American high-school athletes, healthcare professionals were significantly more likely to be present when a concussion was sustained by a boy than a girl, despite girls being more likely to experience a concussion in gender-comparable sports [8].

Beyond the detriment to player welfare, a gender gap in resource-access may also cause a gender gap in the visibility of sporting success. For example, a case study of three co-ed schools observed the provision of spectator buses for boy’s away matches, whereas the girls played their home matches on out-of-town pitches with no spectator transport for home or away games [4]. This gender gap was further exacerbated by the celebration of the boy’s sporting achievements in whole-school assemblies, whilst the girl’s achievements were overlooked [4]. These gender gaps were, in part, enabled by physical education (PE) staff being complicit in the boy’s monopolisation of quality, centre-of-town pitches, and their positioning of the girl’s skills as inherently inferior to the boy’s during mixed-gender PE lessons [4, 5]. Being routinely undervalued within sport may lead to girls internalising such perceptions, identifying as being ‘unsporty’ and subsequently dropping out of teams [4, 9].

Gendered treatment may be particularly prevalent in contact sports, where attributes praised (such as aggression and physicality) conflict with societal expectations of traditional femininity [4, 13]. Indeed, Australian schoolgirls reported a dichotomy whereby women were either *‘tomboys’* or *‘had no ability to play soccer’* [10], whilst Irish girls were denied the opportunity to play rugby as they were ‘too feminine’ and because *‘girls get hurt too easily’* [4]. Perceptions that women have inferior sporting ability compared to men has been observed in player’s families [10], the general public [14], federation presidents [7], and journalists [15]. Indeed, boys are more likely to have parental and peer support during physical activities [16]. Moreover, girls have not only been shown to have less parental support, but are also more likely to have their achievements belittled: *‘you’re good for a girl’* or experience social consequences of sport participation (such as wolf-whistling) [5, 12, 17–19].

For rugby specifically, gendered treatment is frequently reported in the media [20–22] but evidence within peer-reviewed literature remains sparse for adult players. Therefore, the aim of this exploratory study was to interview a range of rugby players to investigate their resource access and the presence of any gender-specific experiences, and enhance our understanding of how different people experience rugby.

## Methods

Ethical approval was granted by the Swansea University FSE Research Ethics Committee (FP_31-03-22b) prior to the start of this study. The study was advertised through social media and email networks, and adult rugby union players (retired or current) in the UK were eligible to volunteer. Recruitment was open between the 16^th^ of May 2022 and the 10^th^ of April 2023. Having provided consent, participants filled in a questionnaire that gathered demographic data and an email address to allow an interview to be arranged. All participants who provided an email address were contacted for interview (n = 51) and 20 (39.2%) participants (ten men, nine women, and one non-binary person aged 29.1 ± 8.3 years; Table 1) completed online interviews that lasted an average of 36 ± 12 minutes.

**Table 1:** Table describing participant characteristics.

Code	Gender	Current Player	Playing environments	Rugby experience (years)
W1	Woman	Yes	Academy/University/Club	10
M2	Man	Yes	Club/University	8
W3	Woman	Yes	Club/University	10
M4	Man	Yes	Academy/University/Club	15
W5	Woman	Yes	University/Club	10
W6	Woman	Yes	University	3
M7	Man	No (retired 10+ years ago)	School/Club/University	25
M8	Man	No (retired 5 years ago)	School/University/Club	11
M9	Man	Yes	Club/University/Semi-professional	17
W10	Woman	Yes	Club	1
M11	Man	Yes	Club/University	17
M12	Man	Yes	School/Club	13
W13	Woman	Yes	Academy/University/Club	17
M14	Man	Yes	Club/Military	30
W15	Woman	Yes	Club/International	11
W16	Woman	Yes	Club	1
M17	Man	Yes	Club/University	30
NB18	Non-binary	Yes	Club	1
W19	Woman	Yes	Club/University	9
M20	Man	Yes	Club/University	19

Online interviews were conducted via Zoom video communication software (Zoom Video Communications Inc, America) to avoid geographical location precluding willing participants from engaging in the study. A semi-structured approach was used, whereby the interviewer followed a question guide (S1 File) to ensure that the questions were relevant to the research aim, but had the flexibility to investigate unique or unexpected personal experiences. The question guide for this study was created in accordance with the semi-structured interview development guide synthesised by Kallio et al. [23].

Pilot interviews were conducted with two men’s and two women’s rugby players and feedback was received on the question wording and interview structure. The interview procedure was then refined to improve question comprehension and allow the interview to flow more naturally (S1 File). Participants were initially asked to describe their journey into rugby, which not only helped to build a rapport but provided insight into potential follow-up questions. Broadly, the interview questions covered three areas: i) how the participants became involved with rugby; ii) their perception of whether their gender has influenced their rugby career; and iii) access to resources throughout their playing career.

Interviews were manually transcribed verbatim and any identifiable participant data were anonymised during transcription by the interviewer at the first opportunity. Transcription was completed simultaneous to data collection, allowing the first author (FJP) to reflect on, and improve the interviews. A six-stage reflexive thematic analysis was then conducted by FJP, in accordance with the procedure described by Braun and Clarke [24]. As there is a dearth of research investigating the lived experiences of rugby players, reflexive thematic analysis was considered the most appropriate analytical process as it allows for the inductive identification of codes and themes [25]. Through this inductive approach, data is not inserted into pre-existing, theory-driven frameworks, rather, novel frameworks are developed from codes that are detected and interpreted as meaningful by the researcher. Themes can be considered ‘domain summaries’ that are produced by grouping inductive codes that share a common core meaning [25].

Having systematically read through the data, initial codes were generated that grouped key information using NVivo software (Version 20, QRSE International), prior to being reflected upon, reviewed, and revised where appropriate. These codes were later aggregated into themes that grouped related codes. Themes were reviewed and the appropriateness of code allocation was checked against the criteria described by Braun and Clarke [26] by FJP and a critical friend. Specifically, the critical friend considered coding decisions and the inclusion parameters of each theme. For retention within the final data set, the coherency and extent to which themes were supported by meaningful data was considered by all the authors.

In rugby, each player may have a different experience that is influenced by their peers, their gender, and/or playing country, thus, in this investigation, reality was not considered singular. This phenomenological study was aligned with the constructivist paradigm, where knowledge is considered to be socially constructed through player-peer and player-environment interactions, rather than received from an external reality or an objective world [27]. It is pertinent to note that using this approach, the researcher’s position (their characteristics and experiences) influences the knowledge produced [25]. FJP is a white British woman, who played university-level rugby between 2016 and 2019. This familiarity with the sport allowed her to build a rapport with participants through shared experiences.

## Results

**Fig 1:**
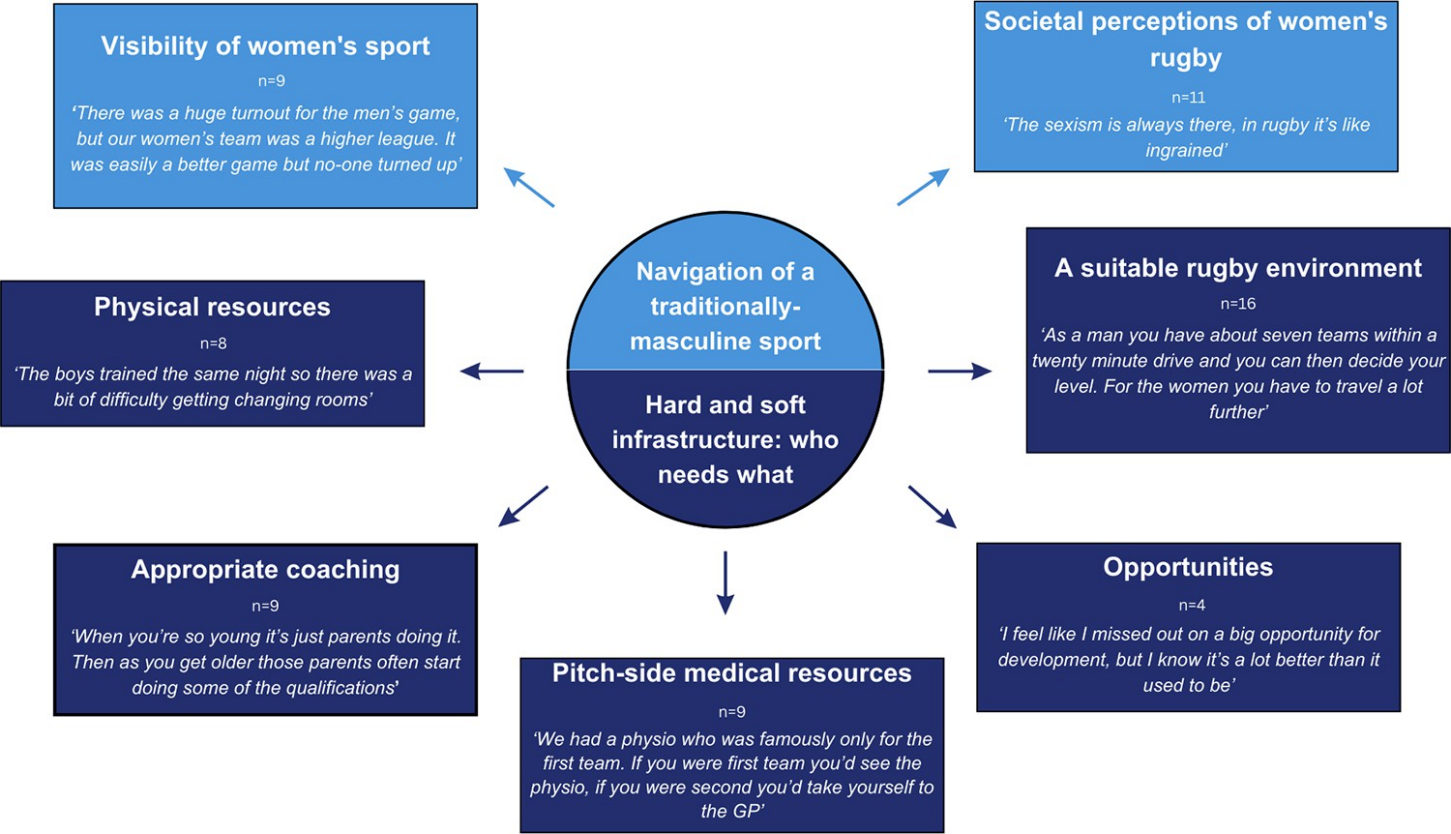
A pen profile detailing codes and themes identified from the interview transcripts. N refers to how many participants discussed each code during interview.

As illustrated in Fig 1, a total of two themes were identified from the interview transcripts. The first theme, Navigation of a traditionally masculine sport, was associated with the codes Visibility of women’s sport and Societal perception of women’s rugby. The second theme, Hard and soft infrastructure: who needs what? was associated with the codes Physical resources, Appropriate coaching, Pitch-side medical resources, Opportunities, and A suitable rugby environment. A circular format was used in the design of the pen profile to acknowledge that each code was a small part of a much wider picture and codes were able to interact and influence each other.

### Navigating a traditionally masculine sport

#### Visibility of the women’s game

Women reported watching their respective men’s team matches, but that this support was often unreciprocated (Table 2, Quotes 1–3). They expressed frustration at this, particularly when providing support to the women’s team was logistically simple: *‘we played just before the men’s game as well*. *They could have just turned up earlier*. *But no’* (W6). Such under-appreciation was reportedly typical of the sport, and that the term rugby was synonymous with men’s rugby (Table 2, Quote 4). Indeed, some men were unaware that their club had a women’s team: *‘I actually found out the other day at a social’* (M12). Participants noted that women players typically ‘*received a lack of recognition’* (W3) and that societally ‘*it was the norm for boys to play rugby*, *whereas the girls don’t really’* (W3). However, these experiences were not universal, as two participants reported mixed-gender fitness sessions and being supported by the men’s teams (Table 2, Quote 5). Men typically perceived that their experience of rugby was enhanced through their gender identity in terms of opportunities to play and access to resources (M2, M7, M14, M20, M11). Such participants noted that the gender gap in rugby was narrowing over time, but further efforts were needed to sustain positive change within the sport.

**Table 2:** Supporting quotes for the theme ’Navigating a traditionally masculine sport’.

Quote	Code	
1	W5	‘There was a huge turnout for the men’s game, but our women’s team was in a higher league. And it was easily a better game, but like no-one turned up but the rest of our team and our mates. The stadium was absolutely packed for the men’s game’
2	M12	‘I’ve never seen the women’s team play’
3	M8	‘The women probably did come to our games a lot more’
4	W19	‘They’ve never heard of women’s rugby; how can you say that you are a fan of rugby? How can you say you love rugby and not know who Sarah Hunter is? If you don’t know anything about the women’s game, you don’t love rugby, you only love men’s rugby’
5	NB18	‘I wasn’t expecting this meeting typical rugby boys, I know it’s a stereotype, but they’ve actually got a lot of respect from our team. It’s genuine compliments after the game. Not airy-fairy things like oh that was good for a lady’s game. Like its genuine. Like you did this really well, our team could work on that’
6	W13	‘They’d done a good six high tackles, near enough in a row. And in a men’s game you think the next person that’s done it that’s a card. But the referee kept giving them a warning. I said is it not time to think about a card and he said, oh well, I don’t want to ruin the game’

#### Societal perceptions of women’s rugby players

Other than negativity associated with the drinking culture within rugby (M2, M4, M12), poor societal perceptions of men’s rugby were not highlighted by participants. Conversely, women highlighted that the women’s game could be viewed poorly by the rugby community and outsiders: *‘unless you’re really keen*, *most people don’t have the best opinion on women’s rugby’* (W6). Further, women reported disparaging comments against women’s rugby made online, and disliked the *‘constant’* (W6) and ‘*unnecessary*’ (W6) judgement of playing level and style made between men and women.

The perception that women were inferior rugby players in comparison to men extended to the conduct of referees. Women reported that their teams were consistently allocated poorer-quality referees: *‘It’s bad*. *I’ve played hundreds of games*. *The standards haven’t been as high for us girls as it has been for guys’* (W3). This poor-quality refereeing was detrimental to player safety: *‘If the referee isn’t as good*, *they don’t call out dangerous moves*, *all those little things can lead to an accumulation of a big injury’* (W13). It was perceived that referees were ‘*less willing to punish players in the women’s game than the men’s’* even when players acted in a dangerous manner (Table 2, Quote 6). Players understood that some leniency could be acceptable when refereeing inexperienced players, but dangerous play should not be tolerated. Participants proposed that women’s rugby was considered a slower, simpler game that was suited to inexperienced or older referees: *‘we’ve had a lot of new referees*, *older referees*, *or they don’t referee men’s rugby anymore*, *they referee women’s because they struggle to keep up’* (W13). Where older referees did not have appropriate physical capabilities, the quality and safety of the game was affected: *‘I’ve had a referee turn up saying he had really bad knees and was walking around the pitch*. *Why are you refereeing then*?*’* (W13).

One woman player who was also a referee, experienced disrespect because of her gender. W19 reported being consistently allocated to women’s games because she *‘would have fun running around with the ladies*.*’* She replied that she would ‘*happily be assigned any game and that she didn’t exclusively referee women’s games*’ but was allocated a women’s game because she *‘was a woman*.*’* For future games, she requested again to be assigned to any game and was then assigned to a gay and inclusive team. She believed that this decision was made on the basis that the fixtures secretary perceived that *‘they’re not a men’s team so they’d be a softer team to referee*.*’* After this negative experience, she formally reported the fixtures secretary but was *‘blacklisted’* and experienced further negativity during her assessments: *‘people would be like oh is she that person everyone is talking about*. *Anytime I was assessed*, *I was assessed as a women’s referee*.*’*

Women typically reported that rugby players were generally supportive of the women’s game (M8, M11, W16, NB18), but that spectators (W5) and club committee members (W6, W3, W5, W19) engaged in sexist behaviours. W19 had *‘contemplated quitting’* after the sexism she had experienced but was driven to change the sport for women in the future: *‘I knew I had to stay*. *Otherwise*, *they’ll never have a female referee*. *I’m quite strong minded and able to endure it so other people don’t have too*.*’*

### Hard and soft infrastructure: who needs what?

Feelings of resentment were shared by women who perceived that they had to tolerate being undervalued in rugby, as negative attitudes were so embedded in the sport: *‘if you’ve got the passion for the sport*, *you just grit your teeth and go with it*. *You get used to it*. *I’m not used to having medical care*, *the big coaches*, *good referees’* (W3). The reasoning suggested for this under-prioritisation was *‘probably just sexism’* (W19). Whilst resentful, W19 expressed such views very casually, suggesting long-term exposure to such sexism, and a reluctant acceptance of a degree of synonymity between rugby and sexism.

#### Physical resources

Women reported poorer access to quality pitches. One participant recalled that her team were consistently allocated *‘out of town’* (W5) pitches with poorer facilities. Consequently, the team’s visibility suffered: *‘the vast majority of people lived in town*, *and unless it was on the good pitch people didn’t come to watch*.*’* The under-prioritisation of women commonly manifested in resource allocation, as exemplified by W19’s experience, where her club ‘*just didn’t want us* (her) *to be prioritised even though we* (they) *were national 15s champions’*. Her team paid the same membership as other teams at her club, but did not get equal access to club provisions: *‘we didn’t get aftermatch food*, *we’d have whatever food was left over from the minis’*, ‘*even if the Astroturf wasn’t being used*, *we’d still train on a patch of grass*.*’* She believed that this inequality was caused by the male-dominated club management and cited that women did not feel comfortable running for these positions (Table 3, Quote 1).

**Table 3:** Participant quotes—hard and soft infrastructure.

Quote	Code	
1	W19	‘Women don’t have that confidence. Men have the arrogance to run for chair, but women would be doing the dirty work. Doing the cafeteria, doing the minis, and they’ll have a far better understanding of the games. It’s quite gendered roles, café, minis, physio’
2	W19	‘It was easier for it to be a men’s changing room. For them it was too much of a hassle to have girls walking around a changing room where men might be’. I never had a changing room at the girls’ teams I played at’
3	W19	‘They’ll come in and go right, we need to learn how to kick, but will have no knowledge of female anatomy. They try to mimic men’s in terms of big, bash-up rugby but we are often better at hands down the line rugby’
4	W1	‘I’ve probably had a lot more opportunities than boys had when I was in the youth game. I got to do under 20s and I probably wasn’t anyway near as good a playing standard. It’s just because you’re in a small and fleeting group you get these opportunities. I also got to do a free refereeing course as they are trying to push for female referees’
5	W13	‘You used to have people grabbing shirts rather than actually making proper tackles. If they were smaller, you’d have more chance of evasion rather than being caught by a shirt billowing behind you’
6	W13	‘If this was a boys’ team, he probably would have got actual kit, probably have been put up in a hotel and probably actually be given some food. It’s the little things like that, you think well this wouldn’t have happened with boys would it’
7	W5	‘We weren’t allowed to play football, we had to play netball. The boys played football and the girls played netball. That was the rules. I definitely wanted to play football’
8	NB18	‘My physical education teacher was really good at wanting it [curricular sport] to be inclusive for girls. She really wanted to promote girls sport, and that girls can do whatever sport they want to do. It was literally two sessions; it was pretty much jump in and do it’
9	M9	‘Most clubs don’t have the same membership for men and women. At home, as a man you have about seven teams within a 20-minute drive and you can then decide your level, but for the women you have to travel a lot further’
10	M4	‘I wasn’t fully developed, but I was playing against boys who probably were. I think the injury risk jumps massively’

Access to changing rooms varied by team. Participant W19, who did not have access to changing rooms, recalled leaving *‘straight after games’* and *‘lost out’* on the social side of rugby (W19). Where changing rooms were present, by default they were allocated to the men (Table 3, Quote 2). This was of detriment to the club’s social community and the likelihood of identifying injuries: *‘You wouldn’t have any connection; you wouldn’t be checking in on people’* (W19). This lack of facilities affected women referees as well: *‘If there was a referee’s changing room*, *I’d change outside because there would be two men referees in there*.*’* Indeed, W19 reported frustration at the club’s response when she was not provided a private changing area: *‘they start getting flustered about where I’m changing*, *as if I’m the problem*.*’* She described the tokenism by which her women’s team were treated: *‘again*, *its men’s clubs with a women’s team*. *They saw it as an opportunity to get funding from the* [relevant rugby union] *but they didn’t really care*. *It was always like that at club* [level].’ Other clubs were *‘proactive’* about creating new women’s facilities: *‘they made sure that we had our own toilets*, *and separation between the men and women’s changing area*.’ Some participants had access to shared changing rooms, but resultantly their privacy could not be guaranteed.

Access to equipment was variable across playing levels. Participants from high-performance university teams reported ‘*unbelievable’* access to resources and were *‘very fortunate’* (W19, M11). M11 described how these resources were provided with an expectation of high performance: *‘in return the demand they asked from you was very high*’. At club level, access to resources was described as *‘pretty good’* (M20), with tackle bags and scrum machines typically available. Some participants reported that access to equipment was through luck or the generosity of other teams rather than access to funding: *‘two clubs that joined into one’*, *‘I’ve got a good relationship with the men’s team*, *I’ve known the secretary a long time’* (W16, NB18).

Where clubs had multiple teams, resources were prioritised towards the first team regardless of gender, and second teams were left to *‘fend for themselves’* (M20). This inequality was said to create a *‘huge divide’* (W13) between players. Resource allocation was also influenced by team attendance. Women’s teams had fewer members, therefore were allocated fewer resources: *‘obviously management go well*, *if you’ve got more boys than girls*, *we’ll give them more time on the scrum machine’* (W3). This was echoed by M9: ‘*there was a lot more engagement from the men’s side*.*’*

#### Access to appropriate coaching

Participants were trained by amateur or professional coaches, dependent on level. During childhood, participants recalled being taught by their own, or teammate’s, fathers who volunteered their time and gradually completed coaching qualifications (M2, M20, M11, W19, M17). The importance of qualified coaches to introduce contact skills was stressed by M14, who questioned the ability of unqualified coaches to teach the *‘correct technique*.*’* However, M14 did acknowledge that *‘standardising the coaching at that level is easier said than done*.*’* One participant expressed that she had experienced *‘a lot of bad practice’* (W19) being coached by volunteers and that the manner of coaching was *‘really inappropriate’* with innuendos being used when coaching young children. School rugby was typically taught by Physical Education teachers, with the exception of private schools that had rugby-specific coaches.

As with other resources, first teams had priority over coach access. Second teams *‘struggled’* accessing coaches as there was less attraction to the role, particularly for lower-level teams: *‘unless you’ve got a volunteer who is willing to go with the second team*, *who’s enthusiastic about getting them to a decent level*, *it’s really hit and miss’* (W15). W13 highlighted that the *‘mentality was still around’* that the women’s game was perceived to be less skilful than the men’s and that coaching was ‘*more interesting with guys’* (W3). Women suggested that coaching men was considered more prestigious than coaching women, regardless of level: *‘one coach was coaching* (a premier club’s) *women’s team*, *then he got offered a promotion* (participant gestures air quotes with their hands) *to the men’s*. *The only time men coach high level women’s is when they want to get to the men’s’* (W19).

Women considered that the translation of training techniques from men to women could be ineffective and that coaches needed to understand that women were not *‘little men’* (Table 3, Quote 3, W19, W13). Conversely, a coach who adapted his delivery of feedback to suit individual players was praised: *‘some people would come off the pitch in tears*, *and he’d wait for them to cool off*, *he fundamentally understood women and understand we worked differently’* (W19). Multiple women reported disrespect from their coaches, whereas no men reported such experiences. W13 was angry that ‘*the head coach kept calling* (her) *different names*’, and because she *‘didn’t feel that the treatment was right’* decided to leave the development team. This lack of respect was detrimental to injury reporting behaviours: *‘if he can’t remember my name*, *I can’t tell him how I’m feeling that day*. *I don’t think I could bring up any injuries or ask him to take me off’* (W19).

#### Access to pitch-side medical resources

Participants playing amateur rugby reported that pitch-side support was opportunistic: *‘anyone medically trained would be roped in’* (M20, M4, M2). Participants considered pitch-side healthcare highly valuable, but persons providing healthcare were not always qualified. Specifically, W15 recalled that her team physio had to leave their club as the union *‘now only allowed qualified physios’*. Whilst the union’s intention behind this ruling was understood, this ‘acting’ physiotherapist *‘was better than nothing’* (W15). Other teams were supported by physiotherapy students from local universities (W15, M17). At schools, first aid was typically provided by teachers who were *‘assumed to be qualified first aiders’* (M4). This first-aid support was considered *‘mostly there for paper purposes’* rather than for the genuine support of injured players (M4). M11 (coaching at a private school) noted that the school outsourced its pitch-side medical support. Pitch-side healthcare was generally less accessible at *‘long distance games as physios didn’t want to give up the whole day’* (W15). Whilst some clubs did employ physiotherapists, second team players expressed that access was ‘*famously only for the first team’* (W6) and resented how injuries were treated differently: *‘if you were first team and got a head knock you told the team physio*, *if you’re second team you went to your doctor and told the coach’* (W6). One woman reported less physiotherapist access: *‘every week the men have a physio and we can’t seem to get one’* (W10).

Physiotherapist presence was considered a necessity, and inaccessibility was cited as a reason for player attrition from clubs: *‘that was one of the reasons we moved*, *the whole team up and left’* (W15). Participants demonstrated a preference for a consistent physiotherapist (W6, M9, W3), who would better understand the player’s injury history and could provide more individualised treatment. Their recovery was quicker as they *‘didn’t have to wait for the NHS’* (M9) and could *‘access free treatment twice a week’* (M9).

#### Access to opportunity

Women highlighted that the under-subscription of women’s teams could mean less competition for opportunities (Table 3, Quote 4), but reported gendered treatment in rugby pathways. W13 compared her treatment as a second-team pathway player against that of the first women’s team and the first- and second-men’s team. Whilst travelling to the same tournament, the women’s first team received a paid-for lunch in a hotel, whilst the second team had to sit outside with a packed lunch. She spoke of how *‘undervalued’* she felt and that a *‘divide’* was created between the teams: *‘the first team were embarrassed*.*’* This divide extended to the provision of kit: *‘the first team were given kit*, *but the second team borrowed kit off another women’s club’* and again felt undervalued: *‘it was kind of disheartening for someone that age*, *whereas the boys second team had kit*, *the first team had kit*.*’* She noted that the ill-fitting shirts affected playing style as well (Table 3, Quote 5). She also spoke of a comparison between the equivalent level boys’ team (Table 3, Quote 6). After a final incident where she was asked to fly to a match, but not given any financial support with travel costs, she *‘lost interest’* and ‘*stopped going’*. She then spoke of how she resented this treatment: *‘I feel like I missed out on a big opportunity for development*.*’*

#### Access to a suitable rugby environment

Access to rugby during childhood was gendered; for boys, rugby was often a curricular sport alongside hockey and cricket, whilst the girls played hockey, netball, and tennis (W6). Participants described how *‘most’* boys playing curricular rugby simultaneously participated in community clubs, further developing their skills (M8, M7, M9, M17). At school, gendered access to sport was not limited to rugby (Table 3, Quote 7). Participants who had played mixed-gender rugby in childhood reported being lost from the sport as clubs *‘didn’t have adolescent girls teams’* that under-11s players could progress into: ‘*you would have to go further afield’* (W19), ‘*there was just no way I could do it’* (NB18).

If rugby was experienced in secondary school by women or non-binary persons, it was offered as a one-off session, typically without contact. To facilitate girls’ rugby in school, teachers had to be proactive: *‘she was driven to provide a range of sports for girls’* (Table 3, Quote 8). Introduction of consistent, regular girls’ rugby sessions were precluded by insufficient teaching resources, or a lack of majority interest: *‘the girls were quite girly*, *they didn’t enjoy the physicality*. *It was the majority*, *so we didn’t do any more’* (W10). Some schools offered non-contact girls’ rugby but players ‘*lost interest when they realised that we weren’t going to play any contact’* (W5). Women noted that they *‘would have loved to have played rugby earlier’* (W16) and that the introduction of rugby in childhood would have made new skills *‘easier’* to learn and their playing level would be higher: *‘it’s taken me six years to get where I am*. *If I had that guidance*, *it probably would have taken me two years*. *It definitely has influenced me’* (W3). Access to rugby was highly important to players and influenced major life decisions: *‘I made sure that the university I went to had a team so I could join it’* (W5).

Regardless of gender, individual differences in skill level and desire to play competitive or social rugby influenced which club players wanted to join. Less confident participants specifically picked a club with a second team so that they could *‘learn how to play*, *then potentially go to the first team’* (W15). As reported by multiple participants, amateur teams were currently ‘*struggling for players’* (W15, M17). The *‘rising petrol costs’* and sparsity of clubs in rural areas precluded the *‘sustained levels of commitment you would need to have a team regularly over years and years’* (W15). Rurally, players also missed out on opportunities to progress through rugby pathways: *‘Dad said the only problem is*, *its 40 minutes away*, *you can’t go every week’* (W13), *‘we got a trial and one of us got in but it was too much of a travel commitment’* (M2). Women were also less likely to have a choice of teams (Table 3, Quote 9).

Low numbers often led to players competing beyond their ability: *‘we were struggling for players*, *so we were pulling players up from the second team who ordinarily wouldn’t be anywhere near first team standard*. *It was a necessity*.*’* This mismatch between experience and match level was highlighted across genders. For men, this *‘mismatch’* appeared most prominent in adolescence due to variation in maturation levels and was perceived to increase injury risk (Table 3, Quote 10). Further, this *‘mismatch’* negatively affected player confidence: *‘I was put off by the bigger boys’* (M17) and enjoyment: *‘me and my mate were just getting run straight over’* (M4).

Participants reported that under-skilled players were allocated to team sheets to avoid sanctions from unions: ‘*the punishments* [enforced by unions] *of not playing a game are so high*, *that it encourages people to play inexperienced players*, *in order to not be punished for not being able to field a full-strength team’* (W15). Reportedly, this pressure to field a team was getting *‘more and more*, *even at the lower levels’* (W15). Established players also reported that a lack of game time led to them leaving rugby: *‘every month there would be a competition*, *but in high school that wasn’t the case and that’s why I lost interest*. *We would just be training and that was it’* (M2).

## Discussion

The current study explored player’s experiences of rugby and their associated access to resources. Specifically, women reported that they had less access to resources, infrastructure, and a suitable training environment than men. Although participants recognised that rugby was evolving to become more welcoming of women players, poor societal perceptions of the women’s game were frequently referenced by participants.

The presumption that women rugby players and support staff are less competent than men in equivalent roles observed in this study is congruent with other recent literature which reports similar bias in refereeing and coaching roles [28–30]. Indeed, in the current study and Fittes [29], coaching positions in women’s teams were reportedly considered a *‘stepping-stone’* to the men’s team, rather than as a valuable opportunity in their own right. In order to change these perceptions, one participant felt compelled to continue her work as a referee to defy the prejudice she had experienced and change the narrative for future generations. This need for women to defy the prejudice imposed upon them was shared by men coaches in interview, who openly voiced their bias against women coaches, whilst simultaneously expecting women to maintain strength of character to challenge the gender bias [28]. In line with previous research, the present study highlights how women in contact sport have been positioned as the ‘problem’, rather than the sexism that is widespread within rugby [4, 5, 28, 31].

The current findings suggest that the gender bias in rugby stems from those in club, union, and school management, rather than from players themselves, who are typically welcoming of women in rugby. Whilst efforts have been made to increase the representation of women in rugby management [1], women in such roles have been exposed to extensive sexism and derogatory comments, whilst little is done to prevent the recurrence of such behaviours [32, 33]. Similarly, one participant formally reported the sexist treatment she experienced as a referee but was not protected from further harm, whilst the person responsible experienced no repercussions. Accordingly, there is a need for a culture change, whereby casual and overt sexism is challenged [17], in addition to the increased representation of women in decision making roles.

The visibility of women in rugby clubs was so poor that several participants reported that men’s players were unaware that a women’s team existed at their club. Similarly, poor visibility has been reported in coaching environments, whereby the men’s coach had only become aware of the women’s team when both coaches happened to attend the same course [28]. Even where men’s teams were aware of the existence of women’s teams, visibility often remained poor as women were allocated out-of-town pitches with worse facilities that further positioned women as outsiders [5]. The poor visibility of women’s rugby and the perception that the women’s game is less interesting likely means that spectator attendance at games is lower. In turn, this lower attendance [4] reduces the probability of a medically trained spectator being present, further reducing the access of women to pitch-side healthcare.

Participants reported their father’s involvement within their rugby career as coaches and volunteers, but not their mother’s. Indeed, gendered volunteer roles have been previously reported in sport, whereby men participate in more overt, authoritarian roles, such as coaching or making announcements, whilst women undertake more covert roles, such as fundraising [34, 35]. The children of volunteers were cognisant of these gendered roles and valued ‘men’s’ roles more highly, whilst being less aware of the contributions from women volunteers [34]. Speculatively, this may be true of rugby given the historic and modern-day sexism that women have experienced in playing, managerial and volunteer roles [29, 33, 36, 37].

Gendered access to sport in childhood was highlighted by participants. Boys could not access traditionally feminine sports like netball, whilst girls could not access traditionally masculine sports like rugby. Women resented the gendered access to rugby during their childhood, noting that contact sport was *‘banned for girls’* but curricular for boys. This casual prohibition draws upon parallels of attempted and actual bans of women in football and rugby [38, 39], albeit in a more covert way. Denying girls the opportunities to play contact rugby, whilst facilitating and promoting boy’s rugby, reinforces historic attitudes whereby women’s bodies are associated with fragility and cannot withstand contact sport [4]. This perception that the female body is inherently flawed leads to reductive injury surveillance research, whereby injury is considered from a solely biological perspective without the consideration of the gendered environmental background [31, 40].

In both men and women’s rugby, second team players reported poorer access to resources than first team players. Whilst likely reflective of limited funding, poor allocation of resources may lead to player attrition. Further, participants highlighted the reliance on volunteer labour in community rugby and considered that there was less attraction to roles within second, rather than first teams. Whilst realistic solutions are challenging, a lack of supporting volunteers may speculatively begin a cycle of negative feedback, where low player numbers cause poor performance and volunteers are less likely to engage within a team.

Whilst this investigation highlights key areas where change is needed, there are certain limitations that must be acknowledged. Indeed, this study is exploratory in nature and further research is necessary to understand the extent to which such experiences are shared amongst a more diverse population. Such research would highlight areas that should be prioritised in future interventions to support the sustainable growth of women’s rugby. It is likely that a degree of selection bias is present within this study; however, efforts were made to broaden the accessibility of participation such as conducting online interviews at any time convenient to the participant. Within the current study, there is a degree of irregularity regarding the frequency at which participants are quoted in the text. Whilst care must be taken not to over generalise these findings, the experiences of individuals should not be overlooked, particularly where participants report team-wide issues on behalf of fellow players. This study, therefore, provides important foundations and highlights an urgent need to address the gendered treatment currently present in rugby to protect player welfare.

## Conclusion

Overall, this exploratory study demonstrated that rugby player’s access to resources was variable according to gender and playing level. Women typically had less access to resources compared to equivalent level men’s teams, with such gendered treatment reportedly propagated at managerial level rather than from the players themselves. Furthermore, women’s teams were reportedly viewed less favourably than men’s teams by members of the rugby community and by outsiders. Future research should explore the perceptions of those in managerial positions within rugby to better understand the origins of gendered treatment within the sport, thereby enabling the development of strategies and interventions to address the gender-related inequalities highlighted in this study.

## Supporting information

S1 File Interview guide. (PDF)
