# Supplementary material for: ‘Probably just sexism’- gendered experiences of resource access in rugby
Source: PLoS One. 2024 May 21;19(5):e0303972. doi: 10.1371/journal.pone.0303972 (PMC11108195; doi:10.1371/journal.pone.0303972)
Supplement: S1 File — (PDF) [file pone.0303972.s001.pdf]

Interview guide

1. Participant consent will be obtained prior to the start of the interview, and the interviewer will introduce themselves and explain the purpose of the interview.

   We are conducting these interviews to explore your experiences of rugby and how they may, or may not relate to your gender. Just a reminder that anything you say will be anonymised in any publications, and you are more than welcome to stop the interview at any time without any justification from yourself.

2. The interview then begins by asking the participants:

   -*Please could you tell me about your journey into rugby?*

   The interviewer will take notes on demographics or specific experiences that may be relevant for probing questions later in the interview.

- What made you want to start playing rugby?
- How did you get involved in rugby?
- Did you play any other sports before playing rugby?

- What resources do you have available to you to help improve your performance?
- How valuable to you find these / would you like access to these resources & what do you think is stopping you from getting access?
- What resources do you have available to you to help improve your safety?
- How valuable to you find these / would you like access to these resources & what do you think is stopping you from getting access?
- How do you think things would change if you had access to more healthcare resources?

- Do you think your experience of rugby would be different if you had a different gender identity? If so, how do you think it would be different?
- Are resources shared equally within this club? Why are they shared in this way?
- What else should I know about this area to investigate this well?
